# Do dietary supplements prevent loss of muscle mass and strength during muscle disuse? A systematic review and meta-analysis of randomized controlled trials

**DOI:** 10.3389/fnut.2023.1093988

**Published:** 2023-05-11

**Authors:** Hua Ye, Jia-Ming Yang, Yun Luo, Yi Long, Jia-Hong Zhang, Yan-Biao Zhong, Feng Gao, Mao-Yuan Wang

**Affiliations:** ^1^Gannan Medical University, Ganzhou City, Jiangxi, China; ^2^Department of Rehabilitation Medicine, First Affiliated Hospital of Gannan Medical University, Ganzhou City, Jiangxi, China; ^3^Ganzhou Intelligent Rehabilitation Technology Innovation Center, Ganzhou, Jiangxi, China; ^4^School of Rehabilitation, Capital Medical University, Beijing, China; ^5^Beijing Bo'ai Hospital, China Rehabilitation Research Center, Beijing, China; ^6^Ganzhou Key Laboratory of Rehabilitation Medicine, Ganzhou City, Jiangxi, China

**Keywords:** disuse muscular atrophy, dietary supplements, muscle strength, leg lean mass, meta-analysis

## Abstract

**Objective:**

We performed a systematic review and meta-analysis of existing randomized controlled trials (RCTs) to assess whether dietary supplements can prevent loss of muscle mass and strength during muscle disuse.

**Methods:**

We searched the following databases: PubMed, Embase, Cochrane, Scopus, Web of Science, and CINAHL for RCTs assessing the effect of dietary supplements on disuse muscular atrophy without language and time restrictions. Muscle strength and leg lean mass were used as the primary outcome indicators. Muscle cross-sectional area (CSA), muscle fiber type distribution, peak aerobic capacity and muscle volume were used as secondary outcome indicators. The risk of bias was assessed using the Cochrane Collaboration's Risk of Bias tool. Heterogeneity was tested using the *I*^2^ statistic index. Mean and standard deviation of outcome indicators were extracted from the intervention and control groups to calculate effect sizes and 95% confidence intervals, with the significance level set at *P* < 0.05.

**Results:**

Twenty RCTs were included with a total of 339 subjects. The results showed that dietary supplements had no effect on muscle strength, CSA, muscle fiber type distribution, peak aerobic capacity or muscle volume. But dietary supplements have a protective effect on the lean mass of the legs.

**Conclusion:**

Dietary supplements can improve lean leg mass, but did not show a tendency to have an effect on muscle strength, CSA, muscle fiber type distribution, peak aerobic capacity or muscle volume during muscle disuse.

**Systematic review registration:**

https://www.crd.york.ac.uk/PROSPERO/#recordDetails, identifier: CRD42022370230.

## 1. Introduction

Patients recovering from an illness or injury usually require a period of bed rest or limb immobilization. However even a short period of inactivity can result in a significant loss of skeletal muscle mass and strength (1, 2), which might lead to a longer recovery period and a higher risk of disease recurrence (3–6). Skeletal muscle is the most abundant tissue in the body, and when it atrophies, it affects the metabolism throughout the body, such as reduced insulin sensitivity (7, 8), decreased basal metabolic rate (9), and increased body fat mass (10). These factors will affect body functions and result in more serious health problems. Therefore, it is necessary to take measures to prevent loss of muscle mass and strength during periods of inactivity.

It is well known that exercise is the best way to maintain and increase muscle mass (11–13). However, in many cases, patients are not allowed to exercise, so other methods are needed to prevent skeletal muscle atrophy. Loss of skeletal muscle mass due to muscle disuse is attributed to a long-term imbalance between muscle protein synthesis and catabolic rates. A decrease in the basal muscle protein synthesis rate has been reported following bed rest (14, 15) and limb immobilization (16, 17). Previous studies found that supplementation with nutrients, such as dietary protein and essential amino acids, can reduce muscle loss and increase muscle growth during immobilization and aging by stimulating muscle protein synthesis (18). The growth of skeletal muscle is traditionally referred to as skeletal muscle hypertrophy, which is manifested as an increase in muscle mass, muscle thickness, muscle area, muscle volume, and muscle fiber cross-sectional area (CSA) (19). However, it remains unclear about the effect of dietary supplements on disuse muscular atrophy. Some randomized controlled trials (RCTs) showed that nutritional supplementation protected against skeletal muscle atrophy during disuse (20, 21), while some RCTs have showed the opposite results (22, 23). Therefore, a meta-analysis of existing RCTs is necessary to provide updated evidence on the effectiveness of dietary supplements in the treatment of disuse muscular atrophy.

## 2. Methods

This review follows the recommendations of the Preferred Reporting Items for Systematic Reviews and Meta-Analyses and the Cochrane collaboration for systematic reviews (24). It answers the following research question: Do dietary supplements prevent loss of muscle mass and strength during muscle disuse? The study is registered on PROSPERO, and the registration number is CRD42022370230.

### 2.1. Search strategy

We searched the PubMed, Embase, Cochrane, Scopus, Web of Science, CINAHL databases for RCTs assessing the effect of dietary supplements on disuse muscular atrophy from the year of inception to December 15, 2022 with no language restrictions. The search terms were (disused muscle atrophy OR skeletal muscle disuse atrophy OR muscle disuse OR disuse atrophy OR muscle disuse atrophy OR disuse atrophies OR immobilization OR immobilization-induced atrophy OR bed rest OR bed rests OR rest, bed OR rests, bed) AND (dietary supplements OR diet therapy OR nutrition therapy OR dietary supplement OR supplements, dietary OR dietary supplementations OR supplementations, dietary OR food supplementations OR food supplements OR food supplement OR supplement, food OR supplements, food OR therapy, nutrition OR nutrition OR diet therapies OR therapy, diet) AND (randomized controlled trial OR controlled trial OR clinical trial). The detailed search strategy was shown in Supplementary Table S1. Furthermore, we manually searched the reference lists of eligible studies to identify additional studies.

### 2.2. Study selection

Two authors (J-MY, YL) independently screened and selected the studies, with disagreements resolved by a third author (HY). The inclusion criteria for the studies were based on the PICOS (patients, intervention, comparison, outcomes, and study design) principle (25), as shown below:

*Patients (P):* Healthy subjects who were immobilized lower extremity or on prolonged bed rest according to experimental needs.

*Intervention (I):* Nutritional supplements were available in capsules, tablets, liquid, powder form, or supplements were added to the daily diet. Supplements can include macronutrients, such as proteins, carbohydrates, and fats; and/or micronutrients, such as vitamins.

*Comparison (C):* The control group was regular diet or placebo.

*Outcomes (O):* Muscle strength, leg lean mass, CSA, fiber type distribution (%): type I and type II, peak aerobic capacity and muscle volume.

*Study design (S):* Randomized controlled trial.

Studies were excluded if they met the following criteria:

(1) Individuals were excluded if they had musculoskeletal impairments, tumors, or critically ill, etc. (2) The intervention group provided dietary supplements combined with other physical therapy, or did not include dietary supplements. (3) Outcomes did not include outcome indicators any of interest. (4) The type of studies were not RCTs.

### 2.3. Data extraction

Relevant data were extracted from the included studies: (1) name of first author; (2) year of publication; (3) study population; (4) number of participants in the intervention and control groups; (5) type of dietary supplements; (6) dietary supplement dosage and duration; (7) age, sex and body mass index of study participants; (8) differences mean and standard deviation (SD) of muscle strength, leg lean mass, CSA, fiber type distribution, peak aerobic capacity, and muscle volume between control and intervention groups. If mean and SD of the differences were not available, we first tried to contact the authors. After no response, we extracted the mean and SD of the pre-intervention and post-intervention values for the control and intervention groups. If the data in the article was presented as a picture and no response was received after contacting the author for specific values, the WebPlot Digitizer tool (https://apps.automeris.io/wpd/index.zh_CN.html) was used to extract the mean and SD. Data were extracted independently by two authors (YL, HY), and disagreements were resolved by a third author (J-MY).

### 2.4. Quality assessment of the study

The methodological quality of the included studies was assessed independently by two raters (Y-BZ, YL) using the Physiotherapy Evidence Database (PEDro) scale, with discrepancies resolved via discussion with a third rater (FG). The PEDro scale consists of 11 items encompassing external validity (Item 1), internal validity (Items 2 to 9), and statistical reporting (Items 10 to 11). The items are as follows: (1) eligibility criteria and source, (2) random allocation, (3) concealed allocation, (4) baseline comparability, (5) blinding of participants, (6) blinding of therapists, (7) blinding of assessors, (8) adequate follow-up (>85%), (9) intention-to-treat analysis, (10) between-group statistical comparisons, and (11) reporting of point measures and measures of variability. Items are rated yes or no (1 or 0) according to whether the criterion is clearly satisfied in the study. A total PEDro score is achieved by adding the ratings of Items 2 to 11 for a combined total score between 0 and 10. Studies with scores < 4 are considered poor, 4 to 5 are considered fair, 6 to 8 are considered good and 9 to 10 are considered excellent (26).

In addition, we assessed the quality of each outcome according to the Grading of Recommendations Assessment Development and Evaluation (GRADE) criteria (27), assigning ratings of very low, low, moderate, and high, which involved assessing five domains, including risk of bias, directness of evidence, consistency, precision of results, publication bias.

### 2.5. Risk of bias assessment

The risk of bias for the included RCTs was assessed independently by two authors (J-HZ, YL) using the Cochrane Collaboration Network's Risk of Bias tool (28). The following items were assessed for each study: (1) random sequence generation, (2) allocation concealment, (3) blinding of participants and personnel, (4) blinding of outcome assessment, (5) incomplete outcome data, (6) selective reporting, and (7) other bias. According to the Cochrane Handbook recommendations, each item was classified as low risk, high risk (not fulfilling the criteria) or unclear risk (specific details or descriptions were not reported), and disagreements were discussed with and resolved by a third author (FG).

### 2.6. Data synthesis and statistical analysis

Statistical analysis was performed using Review Manager 5.3 statistical software. The mean and SD of differences, sample size were input into the statistical software. If the difference values were not directly available and after contacting the authors without a response, they were calculated using Equation ➀, where SD (b), SD (f), and SD (d) represent the SD of the baseline, final, and difference values, respectively, with a correlation coefficient R value estimated at 0.8. If the standard error (SE) was provided in the article, after contacting the author to obtain the SD without getting a response, the SD was calculated according to Equation ➁, where n represents the sample size. If the median was provided in the article, the median was considered as the mean value. Heterogeneity was tested using the *I*^2^ statistical indicator, and *I*^2^ > 50% was considered as high heterogeneity (29). If *I*^2^ > 50%, a random-effects model was used; otherwise, a fixed-effects model was used (29, 30). We reported the effect sizes using the weighted mean differences (WMD) or standardized mean differences (SMD) and 95% confidence interval (95% CI). According to the Cochrane Handbook, the choice of WMD and SMD depends on whether the outcome index evaluation criteria are the same, and WMD is chosen when they are the same, while SMD is chosen when they are not. In addition, subgroup analyses were performed to assess whether the results differed across conditions, and sensitivity analyses were performed to assess the robustness of the results.


(1) $$\begin{array}{l} SD\left(d\right)\;=\;\sqrt{{SD\left(b\right)}^{2}\;+\;{SD\left(f\right)}^{2}\;-\;\left(2\;\times \;R\;\times \;SD\left(b\right)\;\times \;SD\left(f\right)\right)} \end{array}$$

(2) $$\begin{array}{l} SD\;=\;SE\sqrt{n} \end{array}$$

## 3. Results

### 3.1. Search results

**Figure 1:**
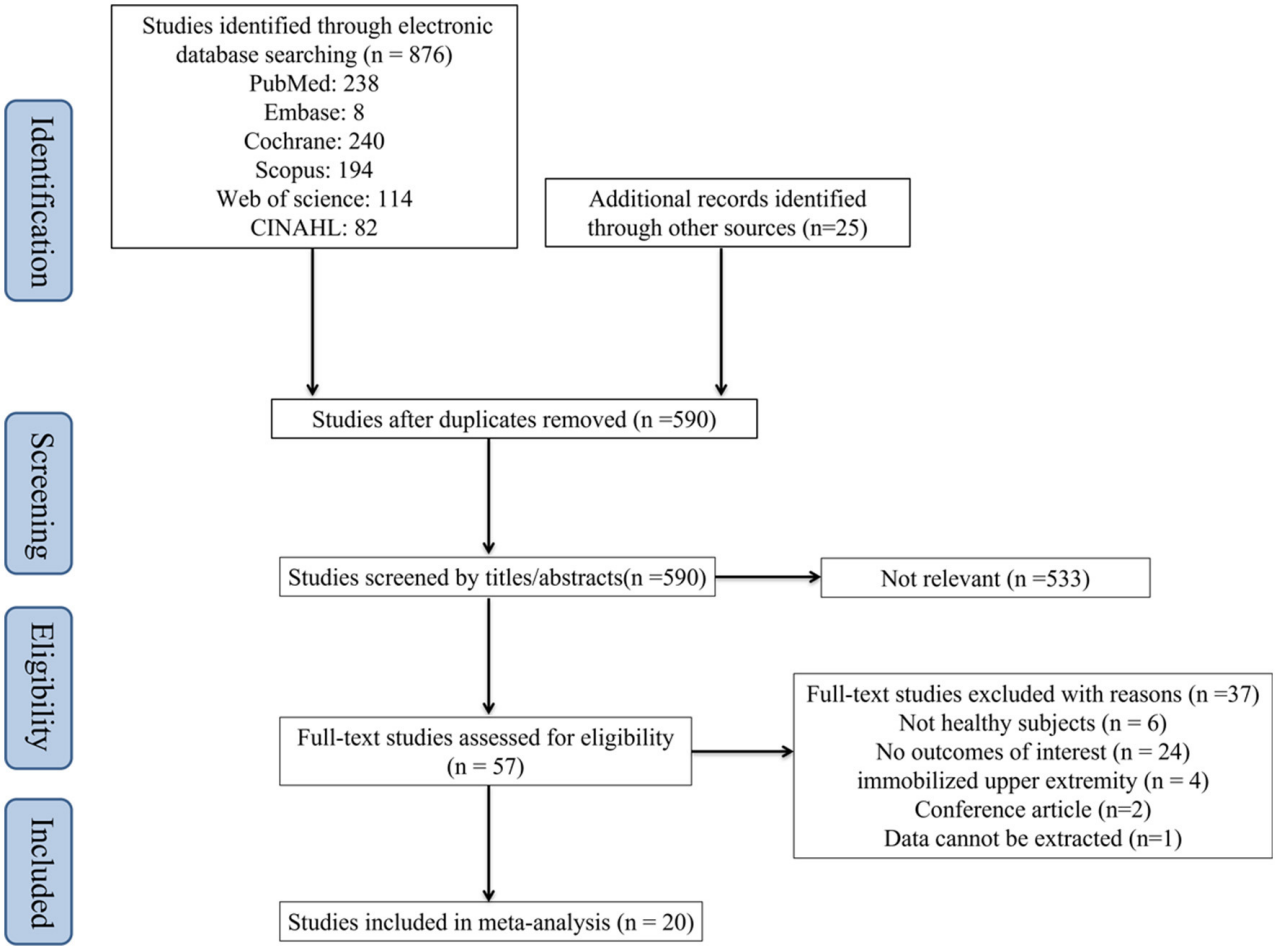
Flow chart of study selection.

The flow chart of study selection was shown in Figure 1. After systematic search from the six databases and other sources, 901 studies were identified. Of which 590 remained after removing duplicates literature. Then, after screening the titles and abstracts of the articles, 533 irrelevant articles were excluded, and 57 were left. Finally, a total of 20 studies (20, 21, 23, 31–47)were included for meta-analysis, and 37 studies were excluded after reading the full article for the following reasons: (1) non-healthy subjects (*n* = 6), (2) no outcome of interest (*n* = 24), (3) conference article (*n* = 2), (4) upper extremity immobilization (*n* = 4), (5) data can't be extracted (*n* = 1). The list of literatures exclusion and reasons for exclusion are shown in Supplementary Table S2.

### 3.2. Characteristics of the included studies

The characteristics of the 20 included RCTs were shown in Table 1. A total of 339 subjects were enrolled, of which about 22% were women, the number of subjects per study ranged from 10 to 30, and their ages ranged from 20 to 70 years. Of the included 20 RCTs, the dietary supplement was leucine in four studies (20, 32, 35, 36), essential amino acids/carbohydrates in the five (41, 43–46), protein in seven (21, 23, 33, 34, 38, 40, 47), creatine in two (31, 37), omega-3 fatty acids in one (39), and β-hydroxy-β-methylbutyrate in one (42). Details of the composition, dose and duration of dietary supplementation performed daily in each study were shown in Table 1. Outcome indicators for only two of the twenty RCTs were expressed as mean and SD (39, 45). The outcome indicators of the other eighteen RCTs were expressed as mean and SE (20, 21, 23, 31–38, 40–44, 46, 47). There are a total of seven studies for the outcome indicator of muscle strength gave the mean and SD of differences (20, 21, 35, 36, 40–42), the eight RCTs did not give, and we calculated SD of differences by the Equation (1) (31–34, 37–39, 46). For the outcome indicator of leg lean mass, there are six RCTs gave the mean and SD of differences (20, 36, 41, 42, 44, 46), and three RCTs did not give (39, 43, 47), we calculate the SD of difference according to Equation (1). For the outcome indicator of CSA, two RCTs had mean and SD of differences (40, 45), and five RCTs were calculated by Equation (1) to obtain the SD of difference (31, 32, 34, 37, 39). For the outcome indicator of muscle fiber distribution, the SD of the difference of all four RCTs was calculated (31–34). For the outcome indicator of peak aerobic capacity, three RCTs gave the mean and SD of differences (20, 21, 36), the SD of difference was calculated for the two RCTs (33, 38). For muscle volume, two RCTs gave the mean and SD of differences (39, 45), the SD of difference was calculated for the two RCTs (23, 38). The data for the six RCTs were presented as pictures in the article, and we extracted the mean and SD by using the WebPlot Digitizer tool (33, 34, 38, 40, 42, 45).

**Table 1:** Characteristics of the studies included in the review.

**Study**	**Disuse muscular atrophy**	**Intervention group**			**Control group**			**Outcomes**	**PEDro Score**
		**Type**	**Dose, duration**	**Population (age, sex, BMI)**	**Type**	**Dose, duration**	**Population (age, sex, BMI)**		
Arentson-Lantz et al. (20)	7 days of bed rest	Leucine	14.6 ± 0.8 g leucine/day	Age (y):68 ± 1 Sex (M/F):7/3 BMI (kg/m^2^):28.0 ± 1.0	Non-EAA alanine	Isoenergetic control alanine: 13.2 ± 0.5 g/day).	Age (y):68 ± 2 Sex (M/F):7/3 BMI (kg/m^2^):25.2 ± 0.7	Knee extensor muscles isometric torque: Nm VO2 peak: L/min Leg lean mass: g	8/10
Arentson-Lantz et al. (21)	7 days of bed rest	Protein	0.90 ± 0.01 g protein/kg/day	Age (y):69 ± 1 Sex (M/F):5/5 BMI (kg/m^2^):27.4 ± 0.8	Isoenergetic diets	-	Age (y):68 ± 2 Sex (M/F):7/3 BMI (kg/m^2^):25.2 ± 0.7	Knee extensor muscles isometric torque: Nm VO_2_ max: L/min	8/10
Backx et al. (32)	7 days immobilization by means of a full leg cast	Leucine	2.5 g leucine, three times daily for 7 days	Age (y):21 ± 1 Sex (M/F):15/0 BMI (kg/m^2^):22.7 ± 0.6	7.5 g maltodextrin and 7.5 g dextrose monohydrate	three times daily for 7 days	Age (y):23 ± 1 Sex (M/F):15/0 BMI (kg/m^2^):23.5 ± 0.8	Knee Extension Strength (1RM): kg CSA: mm^2^ Fiber type distribution (%): type I and type II	9/10
Backx et al. (31)	7 days immobilization by means of a full-leg cast	Creatine	5 g/day creatine for 7 days	Age (y):23 ± 1 Sex (M/F):15/0 BMI (kg/m^2^):23.0 ± 0.5	Maltodextrin 7.5 g and dextrose monohydrate 7.5 g	three times daily for 7 days	Age (y):23 ± 1 Sex (M/F):15/0 BMI (kg/m^2^):23.5 ± 0.9	Knee Extension Strength (1RM): kg CSA: mm^2^ Fiber type distribution (%): type I and type II	7/10
Bosutti et al. (33)	21 days 6° head down-tilt bed rest	Whey protein plus Potassium bicarbonate	0.6 g whey protein/kg body mass/day and 90 mmol KHCO3/day	Age (y):31.6 ± 6.2 Sex (M/F):5/0 BMI (kg/m^2^):23.4 ± 1.6	A basic protein diet	1.2 g protein/kg body mass /day	Age (y):31.6 ± 6.2 Sex (M/F):5/0 BMI (kg/m^2^):23.4 ± 1.6	Knee extensor muscles isometric torque: Nm Fiber type distribution (%): type I and type II VO_2_ max: L/min	7/10
Deutz et al. (42)	10 days of bed rest	β-hydroxy-β-methylbutyrate	3 g/day	Age (y):64 ± 1.4 Sex (M/F):3/8 BMI (kg/m^2^):28.63 ± 4.03	inactive placebo powder	-	Age (y):67.1 ± 1.7 Sex (M/F):1/7 BMI (kg/m^2^):26.5 ± 1.2	Knee extensor muscles isometric torque: Nm Leg lean mass: kg	9/10
Dirks et al. (34)	5 days immobilization by means of a full-leg cast	Protein	20.7 g of protein, 9.3 g of carbohydrate, and 3.0 g of fat twice daily	Age (y):68 ± 1 Sex (M/F):11/0 BMI (kg/m^2^):26.4 ± 0.8	-	-	Age (y):70 ± 1 Sex (M/F):12/0 BMI (kg/m^2^):27.3 ± 0.6	Knee extension strength (1RM): kg CSA: mm^2^ Fiber type distribution (%): type I and type II	6/10
Edwards et al. (35)	7 days immobilization by means of a full-leg cast	Leucine	15 g leucine/d for 7 days	Age (y):22 ± 1 Sex (M/F):8/0 BMI (kg/m^2^):23.8 ± 0.83	Nonessential EAA	15 g placebo/d (non-EAA) for 7 days	Age (y):23 ± 1 Sex (M/F):8/0 BMI (kg/m^2^):22.3 ± 0.9	Knee extensor muscles isometric strength: N	9/10
English et al. (36)	14 days of bed rest	Leucine	0.06 g/kg /meal, three times daily	Age (y):51 ± 1 Sex (M/F):6/4 BMI (kg/m^2^):24.6 ± 0.9	Alanine control	-	Age (y):52 ± 1 Sex (M/F):6/3 BMI (kg/m^2^):24.7 ± 1.7	Knee extensor muscles isometric torque: Nm VO_2_ peak: L/min Leg lean mass: kg	9/10
Ferrando et al. (43)	10 days of bed rest	EAA	15 g of EAA/day	Age (y):71 ± 6 Sex (M/F):1/9 BMI (kg/m^2^): -	A placebo	-	Age (y):68 ± 5 Sex (M/F):6/6 BMI: (kg/m^2^):	Leg lean mass: kg	5/10
Fitts et al. (44)	28 days of bed rest	EAA and carbohydrate	16.5 g EAA and 30 g sucrose thrice daily	Age (y):36 ± 4 Sex (M/F):5 BMI: (kg/m^2^): -	Only the diet soft drink	-	Age (y):38 ± 3 Sex (M/F):5 BMI (kg/m^2^):	Leg lean mass: g	5/10
Hespel et al. (37)	14 days immobilization by means of a full-leg cast	Creatine	5 g of creatine monohydrate four times per day	*n =* 11	Only maltodextrin containing citrate (40 mg /g)		*n =* 11	CSA:cm^2^ knee extensor muscles isometric torque: Nm	9/10
Holloway (45)	7 days of immobilization by means of a full-leg cast	a proprietary EAA	23.7 g EAA, thrice daily	*n =* 10	an excipient- and energy- matched placebo (maltodextrin) liquid drink	-	*n =* 10	CSA: mm^2^ Muscle Volume: mm^3^	8/10
Kilroe et al. (38)	3 days immobilization by means of a full-leg cast	Protein	1.6 g/kg body mass /day	Age (y):22 ± 1 Sex (M/F):11/0 BMI (kg/m^2^):23 ± 1	Protein	0.15 g/kg body mass /day	Age (y):20 ± 1 Sex (M/F):11/0 BMI (kg/m^2^):23 ± 1	Knee extensor muscles isometric torque: Nm VO_2_peak: mL/kg/min muscle volume: cm^3^	7/10
McGlory et al. (39)	14 days immobilization by means of a full-leg cast	Omega-3 fatty acid	5 g/d of n-3 fatty acid	Age (y):22 ± 3 Sex (M/F):0/11 BMI (kg/m^2^):23.1 ± 2.1	Isoenergetic quantity of sunflower oil		Age (y):22 ± 3 Sex (M/F):0/9 BMI (kg/m^2^):23.9 ± 2.5	Knee extensor muscles isometric torque: Nm CSA: mm^2^ Leg lean mass: g muscle volume: cm^3^	10/10
Mitchell et al. (40)	14 days immobilization by means of a full-leg cast	protein	Once daily: 20g milk protein concentrate	Age (y):51.5 ± 3.8 Sex (M/F):15/0 BMI (kg/m^2^):27.5 ± 3.2	Once daily: isoenegetic placebo	-	Age (y):48.5 ± 2.4 Sex (M/F):15/0 BMI (kg/m^2^):28.3 ± 3.2	Knee extensor muscles isometric torque: Nm CSA: mm^2^	10/10
Paddon-Jones et al. (41)	28 days of bed rest	EAA/carbohydrate supplement	16.5 g EAA and 30 g carbohydrate per day	Age (y):36 ± 10 Sex (M/F):7/0	Placebo	-	Age (y):38 ± 8 Sex (M/F):6/0	Knee Extension Strength (1RM): kg Leg lean mass: g	6/10
Paddon-Jones et al. (46)	28 days of bed rest	EAA and carbohydrate	16.5 g EAA and 30 g sucrose	Age (y):36 ± 10 Sex (M/F):7/0 BMI (kg/m^2^):	The diet soft drink	-	Age (y):38 ± 8 Sex (M/F):6/0 BMI (kg/m^2^): -	Leg lean mass: g Knee Extension Strength (1RM): kg	5/10
Rudwill et al. (47)	21 days of bed rest	High-protein intake, (33% whey protein)	1.8 g/kg/day	Age (y):31 ± 2.1 Sex (M/F):9/0 BMI (kg/m^2^):23.9 ± 0.5	Isocaloric control diet	-	Age (y):31 ± 2.1 Sex (M/F):9/0 BMI (kg/m^2^):23.8 ± 0.5	Leg lean mass: kg	5/10
Trappe et al. (23)	60 days of bed rest	protein and free leucine	1.45 g/kg body weight /day + 3.6 g/ day of free leucine	Age (y):29 ± 1 Sex (M/F):0/8 BMI (kg/m^2^): -	Protein	1.0 g/kg body weight/day	Age (y):34 ± 1 Sex (M/F):0/8 BMI (kg/m^2^):	Muscle volume: cm^3^	5/10

BMI, Body Mass Index; M/F, Male/Female;1RM, one-repetition maximum; CSA, muscle cross-sectional area; VO_2_ peak, peak aerobic capacity; EAA, Essential amino acid.

### 3.3. Measurement of outcome indicators

In total, fifteen RCTs measured muscle strength as an outcome indicator. Muscle strength was measured in two ways. Ten RCTs measured knee extensor muscles isometric torque by isokinetic dynamometry (20, 21, 33, 35–40, 42), while the other five RCTs measured knee extension strength by performing one repetition of the maximum strength test on the leg extension machine (31, 32, 34, 41, 46). For the measurement of the lean mass of the legs were all used dual-energy x-ray absorptiometry (20, 36, 39, 41–44, 46, 47). Seven studies reported CSA as an outcome indicator, three of which used CT for detection (31, 32, 34), three studies used MRI for detection (37, 39, 45), and one was detected using Stratec XCT 3000 pQCT with software version 6.20C (40). Outcome indicators of fiber type distribution were taken from the vastus lateralis muscle and then muscle biopsy was performed (31–34). Peak muscle aerobic capacity for the four RCTs was measured by a graded exercise test on a cycle ergometer and metabolic cart (20, 21, 33, 36). Another RCT measure of peak aerobic capacity was the single-leg ramp exercise test (38). The muscle volume was all measured by MRI (23, 38, 39, 45).

### 3.4. Quality of the studies

The methodological quality of the included studies was shown in Table 1 and Supplementary Table S3. Eight studies (20, 21, 31, 33, 34, 38, 41, 45) were rated as good quality. 7 studies (32, 35–37, 39, 40, 42) were rated as excellent quality, and remaining 5 articles were rated fair quality (23, 43, 44, 46, 47). The results indicated insufficient level of concealed allocation, and the sample sizes included in the study were small. The GRADE evidence for the outcome indicators was rated as low, as detailed in Table 2.

**Table 2:** GRADE evidence profile for outcomes among trials included in the systematic review.

**No. of studies (participants)**	**Design**	**Risk of bias**	**Inconsistency**	**Indirectness**	**Imprecision**	**Other considerations**	**Absolute effect**	**Quality**	**Importance**
**Muscle strength**									
15 (299)	RCTs	Serious^*^	No serious inconsistency	No serious indirectness	Serious^†^	None	SMD 0.19 higher (0.05 lower to 0.42 higher)	Low	Critical
**Leg lean mass**									
9 (157)	RCTs	Serious^*^	No serious inconsistency	No serious indirectness	Serious^†^	None	MD 0.39 higher (0.2 to 0.58 higher)	Moderate	Critical
**CSA**									
7 (170)	RCTs	Serious^*^	No serious inconsistency	No serious indirectness	Serious^†^	None	SMD 0.1 higher (0.2 lower to 0.41 higher)	Low	Important
**Type I muscle fiber distribution (%)**									
4 (88)	RCTs	Serious^*^	No serious inconsistency	No serious indirectness	Serious^†^	None	WMD 1 lower (4.58 lower to 6.58 higher)	Low	Important
**Type II muscle fiber distribution (%)**									
4 (88)	RCTs	Serious^*^	No serious inconsistency	No serious indirectness	Serious^†^	None	WMD 1.27 lower (4.92 lower to 2.37 higher)	Low	Important
**Peak aerobic capacity**									
5 (91)	RCTs	Serious^*^	No serious inconsistency	No serious indirectness	Serious^†^	None	SMD 0.03 lower (0.44 lower to 0.39 higher)	Low	Important
**Muscle volume**									
4 (78)	RCTs	Serious^*^	No serious inconsistency	No serious indirectness	Serious^†^	None	MD 62.85 lower (78.28 to 47.42 lower)	Low	Important

^*^Inadequate allocation concealment; ^†^The sample size was too small (*n* < 400).

### 3.5. Risk of bias of the studies

**Figure 2:**
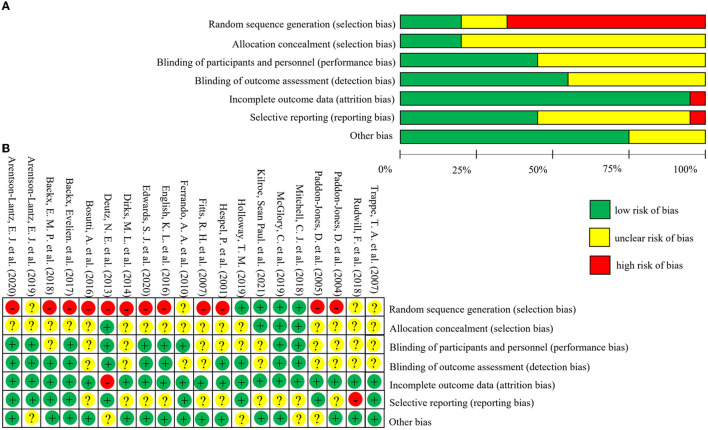
Risk of bias graph and summary of included studies. The figure **(A)** (risk of bias graph) shows the overall risk of bias in each domain. The figure **(B)** (risk of bias summary) indicates the risk of bias in each domain for each study.

The risk of bias of the 20 included studies was shown in Figure 2. Twelve studies were rated as high risk of bias for selection bias because they only mentioned the randomization but did not specify which method was used for random assignment (20, 31–37, 41, 42, 44, 46). Information on allocation concealment was not available in sixteen studies and was therefore rated as unclear risk of bias (20, 21, 23, 31–37, 41, 43–47). Most of the studies on performance bias and detection bias were described as double-blinded and therefore rated as low risk, while those not described were rated as unclear risk of bias. All studies had follow-up rates above 85%, so attrition bias was rated as low risk of bias. Nine studies' reporting bias were rated as low risk because the study protocols were consistent with the outcome indicators in the studies (20, 21, 23, 31, 32, 41–43, 45). And ten studies were rated as unclear risk of bias when there is no enough information about selective reporting (33–40, 44, 46). Other risks of bias that were not mentioned or could not be judged according to the study and were rated as unclear risks. The results of the sensitivity analysis were shown in Supplementary Figure S3.

### 3.6. Effect of dietary supplements on muscle strength in disuse muscular atrophy

**Figure 3:**
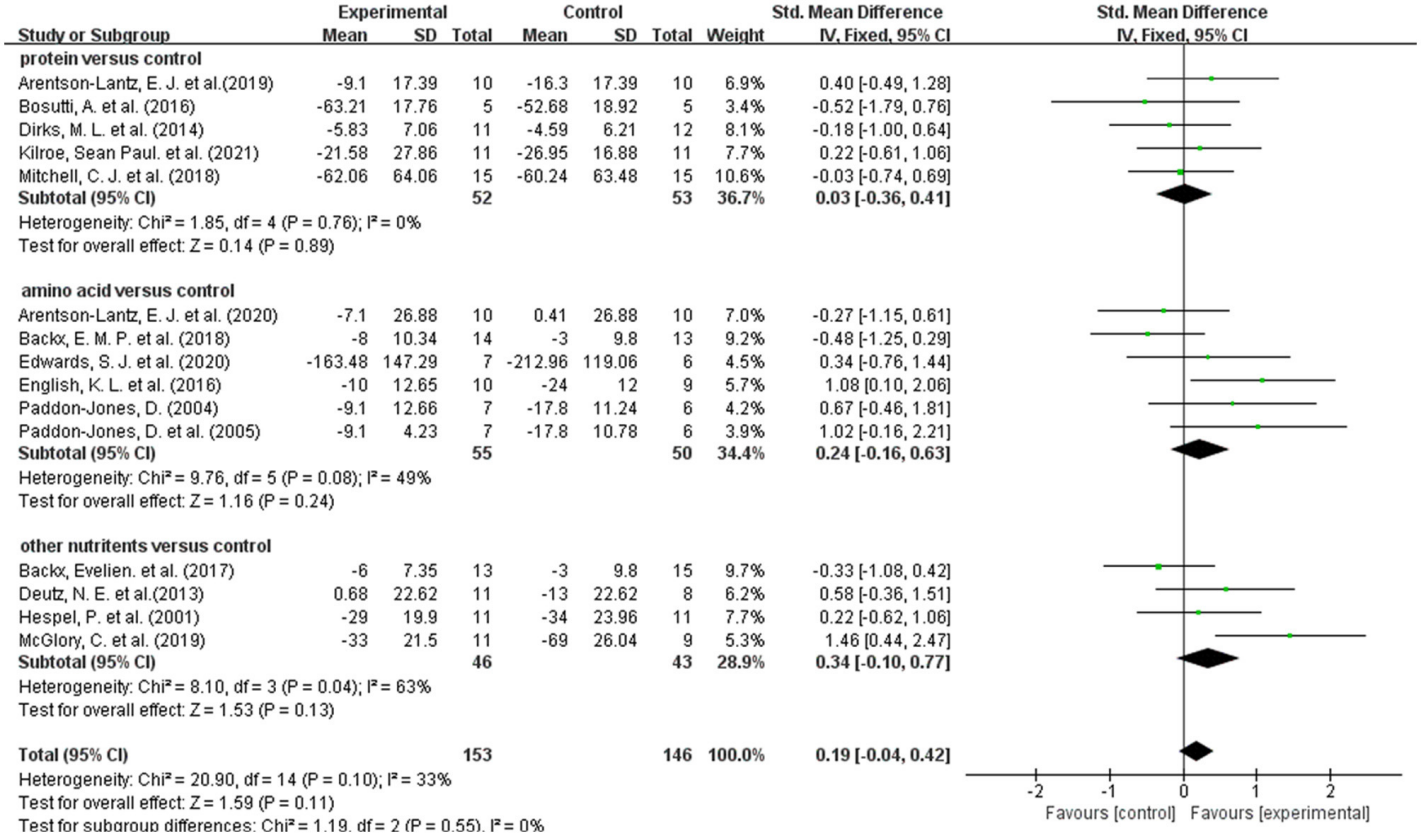
Forest plot of subgroup analysis of SMD difference and 95% confidence intervals for the effect of dietary supplements on muscle strength according to the type of experimental group.

A total of 15 studies measured muscle strength (20, 31–41). Overall meta-analysis showed that dietary supplements had no protective effect on muscle strength during muscle disuse (SMD: 0.19; 95% CI:−0.04, 0.42; *p*: 0.11). As the types of dietary supplements in both intervention group and control group were heterogenous among the included studies, we performed subgroup analysis to test the effect of different types of dietary supplements on muscle strength. Firstly, we divided the dietary supplements in the intervention group into protein, amino acid, and other nutrients. Other nutrients include β-hydroxy-β-methylbutyric acid, creatine and Omega-3 fatty acids. The results of subgroup analysis (Figure 3) showed that neither protein (SMD: 0.03; 95% CI: −0.36, 0.41; *p*: 0.89), amino acids (SMD: 0.24; 95% CI: −0.16, 0.63; *p*: 0.24) nor other nutrients (SMD: 0.34; 95% CI: −0.10, 0.77; *p*: 0.13) had a protective effect on muscle strength. In addition, we performed a subgroup analysis based on the use of placebo and non-placebo in the control group. Supplementary Figure S1 showed that the use of different supplement types in the control group had no effect on the results.

### 3.7. Effect of dietary supplements on leg lean mass in disuse muscular atrophy

**Figure 4:**
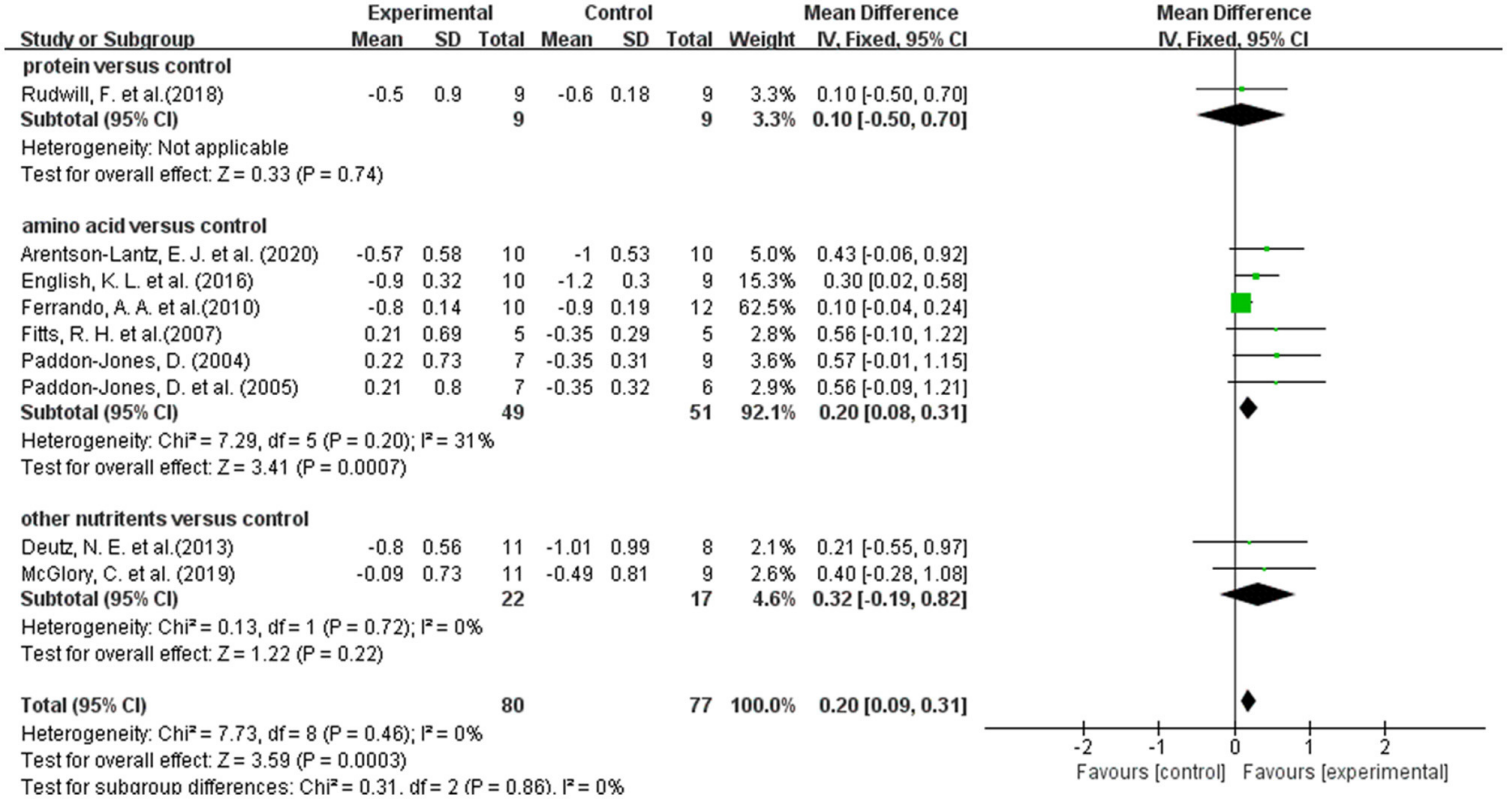
Forest plot of subgroup analysis of WMD difference and 95% confidence intervals for the effect of dietary supplements on leg lean mass according to the type of experimental group.

A total of 9 studies reported on leg lean mass as an outcome indicator (20, 36, 39, 41–44, 46, 47). Overall meta-analysis showed that dietary supplements had protective effect on leg lean mass during muscle disuse (WMD: 0.20; 95% CI: 0.09, 0.31; *p*: = 0.0003). As the types of dietary supplements in both intervention group and control group were heterogenous among the included studies, we performed subgroup analysis to test the effect of different types of dietary supplements on leg lean mass. We divided the dietary supplements in the intervention group into protein, amino acid, and other nutrients. But the protein group had only one article and meta-analysis was not possible (47). The results of subgroup analysis (Figure 4) showed that the amino acid group significantly improved the lean mass of the legs (WMD: 0.20; 95% CI: 0.08, 0.31; *p*: = 0.0007), but the other group did not show an effect (WMD: 0.32; 95% CI: −0.19, 0.82; *p*: 0.22). In addition, we performed a subgroup analysis based on the use of placebo and non-placebo in the control group. Supplementary Figure S2 showed that the use of different supplement types in the control group had no effect on the results.

### 3.8. Effect of dietary supplements on secondary outcome indicators in disuse muscular atrophy

#### 3.8.1. CSA and muscle fiber type distribution

**Figure 5:**
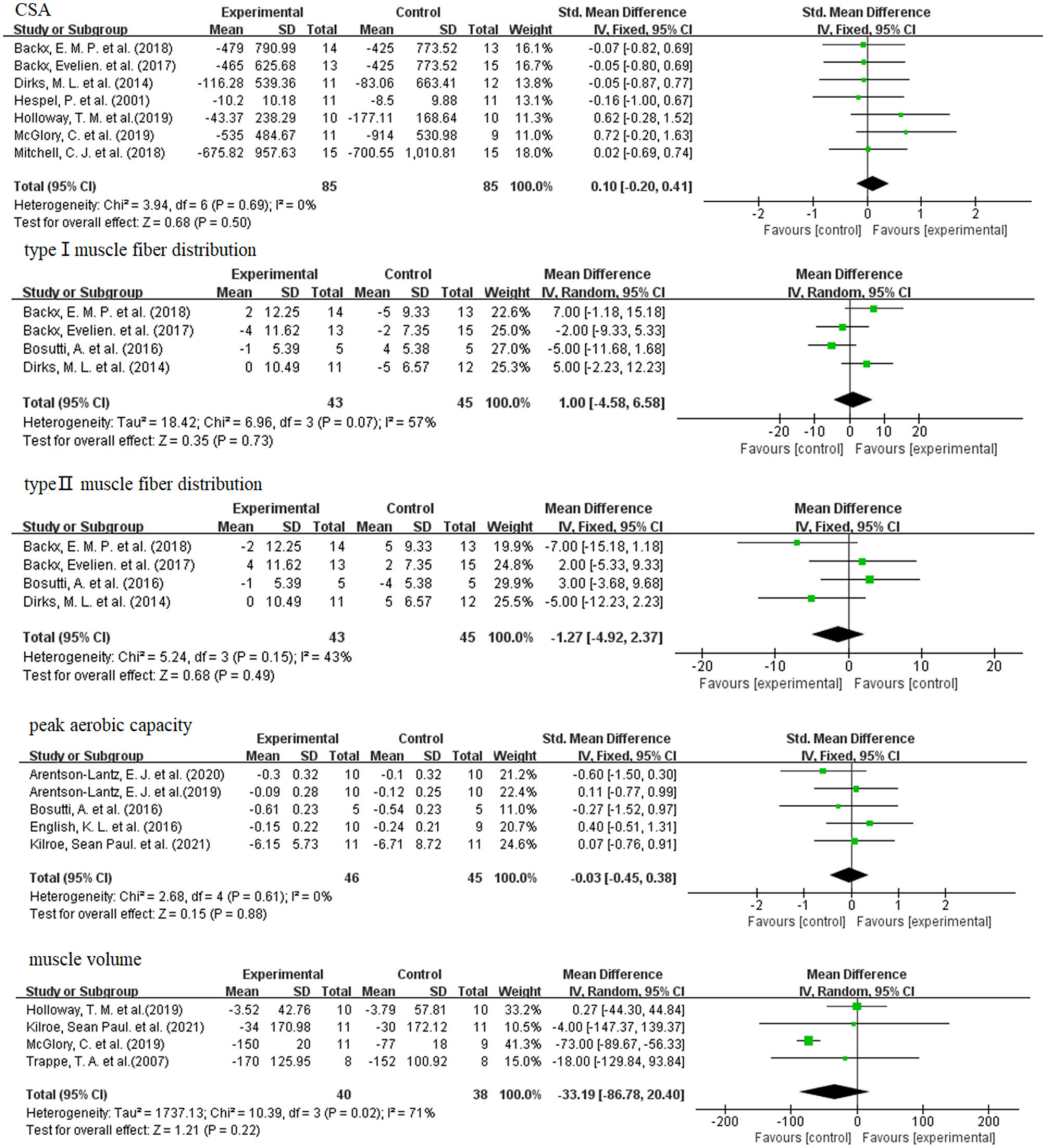
Forest plots of SMD, WMD and 95% confidence intervals for the effect of dietary supplements on CSA, type I muscle fiber distribution (%), and type II muscle fiber distribution (%), peak aerobic capacity, and muscle volume.

The results of the meta-analysis of CSA and muscle fiber type distribution are shown in Figure 5. When skeletal muscle atrophy occurs, the CSA becomes smaller. Dietary supplement group did not exhibit greater CSA compared to the control group (SMD: 0.10; 95% CI: −0.20, 0.41; *p*: 0.55). When skeletal muscle atrophy occurs, type I muscle fibers are converted to type II muscle fibers (48, 49). And then the distribution of type I and type II muscle fibers was altered. As shown in Figure 5, dietary supplementation had no effect on the distribution of type I (WMD: 1.00; 95% CI: −4.58, 6.58; *p*: 0.73) and type II (WMD: −1.27; 95% CI: −4.58, 6.58; *p*: 0.49) muscle fibers.

#### 3.8.2. Peak aerobic capacity

When skeletal muscle atrophy occurs, the peak aerobic capacity of the muscle becomes poor. However, as shown in Figure 5, meta-analysis showed that dietary supplementation did not improve the peak aerobic capacity of the disuse muscular atrophy process (SMD: −0.03; 95% CI: −0.45, 0.38; *p*: 0.88).

#### 3.8.3. Muscle volume

As shown in Figure 5, meta-analysis showed that dietary supplements had no effect on muscle volume during muscle disuse (WMD: −33.19; 95% CI: −86.78, 20.40; *p*: 0.22).

## 4. Discussion

In this systematic review and meta-analysis, we included 20 RCTs with a total of 339 subjects to analyze whether dietary supplements prevent loss of skeletal muscle mass and strength during muscle disuse. The results of the meta-analysis showed that dietary supplements had no effect on muscle strength, but could improve the lean mass of the legs. For the two primary outcome indicators, muscle strength and lean mass, we performed subgroup analysis based on different types of supplements in the control and intervention groups. The results of all subgroup analyses of muscle strength showed that protein, amino acids and other dietary supplements did not improve muscle strength during muscle disuse. Subgroup analyses of leg lean mass showed that the amino acid group significantly improved lean leg mass during muscle disuse, but the other group did not show an effect. However, their total result was that dietary supplements could improve leg lean mass during muscle disuse. This may be related to the relatively small number of studies in the other group. Our results did not support dietary supplementation had effect on secondary outcome indicators including CSA, muscle fiber type distribution, peak aerobic capacity and muscle volume.

Transient muscle disuse after injury or during recovery from disease results in a loss of muscle mass and function, including a progressive decrease in muscle strength, lean muscle mass, muscle volume, aerobic capacity, CSA, atrophy of type I muscle fibers and a shift from type I to type II muscle fibers (50). It is accompanied by many other negative health consequences, such as reduced insulin sensitivity (7, 8), decreased basal metabolic rate (9), and increased body fat mass (10). Therefore, it is important to find a safe and effective measure to alleviate skeletal muscle atrophy during muscle disuse. Our findings suggest that dietary supplementation can play a role in maintaining lean body mass during muscle disuse. Dietary supplements may be considered to maintain lean muscle mass when patients are unable to exercise during muscle disuse. Overall, the risk of bias in our included RCTs was low, and the heterogeneity of the outcome indicators was small. Therefore, our conclusions may provide some guidance for clinical practice.

To our knowledge, this is the first systematic review and meta-analysis of the role of nutritional interventions on disuse muscular atrophy. Our results are consistent with previously published systematic reviews of nutritional interventions for elderly or sarcopenic patients, where nutritional supplementation alone had no effect on muscle strength (51–53). This may be related to the fact that muscle strength is determined by many factors, such as neuromuscular control and muscle mass. However, studies have found that nutritional interventions combined with exercise training can improve muscle strength and physical function (53–56). Therefore, we recommend that nutritional supplementation combined with exercise training interventions should be performed to improve muscle strength after the release of exercise contraindications. For lean mass, the results of our meta-analysis are inconsistent with previously published systematic reviews of nutrition interventions in older adults (52, 57), which may be related to the different populations we included. Our systematic review included healthy subjects, whereas their systematic review included older adults, whose body composition would have changed.

However, this study has some limitations. Firstly, the sample sizes of the included studies in the systematic review were relatively small. Secondly, the number of studies on outcome indicators such as CSA, muscle fiber type distribution, peak aerobic capacity and muscle volume was relatively small. Third, the number of studies for subgroup analysis was also small. Fourth, some of the RCTs we included do not directly give the SD of the difference, which we calculated by the formula and may differ from the actual value. In addition, some of the outcome indicators are presented in the form of pictures, and the results we obtained by using the WebPlot Digitizer tool to extract the mean and SD may also be different from the actual values. Fifth, the types of dietary supplements in both intervention group and control group were heterogenous among the included studies. Therefore, more RCTs with larger sample sizes are needed in the future to validate the effects of dietary supplements on these outcome indicators.

## 5. Conclusion

Dietary supplements can improve the lean mass of the legs during muscle disuse. However, our results do not support the role for dietary supplements on muscle strength, CSA, muscle fiber type distribution, peak aerobic capacity or muscle volume.

## Data availability statement

The original contributions presented in the study are included in the article/Supplementary material, further inquiries can be directed to the corresponding authors.

## Author contributions

Conception and design and drafted the manuscript: HY, J-MY, and Y-BZ. Collection and analysis of the data: YLu, YLo, and J-HZ. Final approval of the article: FG and M-YW. All authors contributed to the article and approved the submitted version.

## Supplementary material

Supplementary Figure S1 Forest plot of subgroup analysis of SMD difference and 95% confidence intervals for the effect of dietary supplements on muscle strength according to the type of control group.

Supplementary Figure S2 Forest plot of subgroup analysis of SMD difference and 95% confidence intervals for the effect of dietary supplements on leg lean mass according to the type of control group.

Supplementary Figure S3 Results of sensitivity analysis on muscle strength, leg lean mass, CSA, type I muscle fiber distribution (%), and type II muscle fiber distribution (%), peak aerobic capacity, and muscle volume.
