# Supplementary material for: Do dietary supplements prevent loss of muscle mass and strength during muscle disuse? A systematic review and meta-analysis of randomized controlled trials
Source: Front Nutr. 2023 May 11;10:1093988. doi: 10.3389/fnut.2023.1093988 (PMC10210142; doi:10.3389/fnut.2023.1093988)
Supplement: Supplementary file 1 [file Table_1.DOCX]

Table S1. Databases search process.

| Databases | PubMed, Embase, Cochrane, Scopus, Web of Science, CINAHL |
| --- | --- |
| Steps | Search terms for query |
| #1 | (dietary supplements) OR (diet therapy) OR (nutrition therapy) OR (dietary supplement) OR (supplements, dietary) OR (dietary supplementations) OR (supplementations, dietary) OR (food supplementations) OR (food supplements) OR (food supplement) OR (supplement, food) OR (supplements, food) OR (therapy, nutrition) OR (nutrition) OR (diet Therapies) OR (therapy, diet) |
| #2 | (disused muscle atrophy) OR (skeletal muscle disuse atrophy) OR (muscle disuse) OR (disuse atrophy) OR (muscle disuse atrophy) OR (disuse atrophies) OR (immobilization) OR (immobilization-induced atrophy) OR (bed rest) OR (bed Rests) OR (rest, Bed) OR (rests, Bed) |
| #3 | (randomized controlled trial) OR (controlled trial) OR (clinical trial) |
| #4 | #1 AND #2 AND #3 |
