# Supplementary material for: Do dietary supplements prevent loss of muscle mass and strength during muscle disuse? A systematic review and meta-analysis of randomized controlled trials
Source: Front Nutr. 2023 May 11;10:1093988. doi: 10.3389/fnut.2023.1093988 (PMC10210142; doi:10.3389/fnut.2023.1093988)
Supplement: Supplementary file 2 [file Table_2.docx]

Table S2. Studies were excluded after reading the full article for the following reasons.

| Reason | Number |
| --- | --- |
| non-healthy subjects | 6 [1-6] |
| no outcome of interest | 24 [7-30] |
| conference article | 2 [31, 32] |
| upper extremity immobilization | 4 [33-36] |
| data cannot be extracted | 1(n = 1) [37] |

References:

1. Bellanti, F., et al., *An open-label, single-center pilot study to test the effects of an amino acid mixture in older patients admitted to internal medicine wards.* Nutrition (Burbank, Los Angeles County, Calif.), 2020. **69**.

2. Dresen, E., et al., *Medical high-protein nutrition therapy and loss of muscle mass in adult ICU patients: A randomized controlled trial.* Clin Nutr, 2021. **40**(4): p. 1562-1570.

3. Ekinci, O., et al., *Effect of Calcium β-Hydroxy-β-Methylbutyrate (CaHMB), Vitamin D, and Protein Supplementation on Postoperative Immobilization in Malnourished Older Adult Patients With Hip Fracture.* Nutrition in Clinical Practice, 2016. **31**(6): p. 829-835.

4. Gade, J., et al., *Protein-enriched, milk-based supplement to counteract sarcopenia in acutely ill geriatric patients offered resistance exercise training during and after hospitalisation: study protocol for a randomised, double-blind, multicentre trial.* Bmj Open, 2018. **8**(2).

5. Gonzalez, J.T., et al., *Intermittent versus continuous enteral nutrition attenuates increases in insulin and leptin during short-term bed rest.* European Journal of Applied Physiology, 2020. **120**(9): p. 2083-2094.

6. Smoliner, C., et al., *Effects of food fortification on nutritional and functional status in frail elderly nursing home residents at risk of malnutrition.* Nutrition, 2008. **24**(11/12): p. 1139-1144.

7. Hackney, K.J., S.B. Cook, and L.L. Ploutz-Snyder, *Nutrition and resistance exercise during reconditioning from unloading.* Aviation space and environmental medicine, 2011. **82**(8): p. 805‐809.

8. Reidy, P.T., et al., *Neuromuscular electrical stimulation and protein during bed rest increases CD11b+ skeletal muscle macrophages but does not correspond to muscle size or insulin sensitivity.* Applied Physiology, Nutrition & Metabolism, 2020. **45**(11): p. 1261-1269.

9. Standley, R.A., et al., *Effects of β-hydroxy-β-methylbutyrate on skeletal muscle mitochondrial content and dynamics, and lipids after 10 days of bed rest in older adults.* J Appl Physiol (1985), 2017. **123**(5): p. 1092-1100.

10. Op 't Eijnde, B., et al., *Effect of oral creatine supplementation on human muscle GLUT4 protein content after immobilization.* Diabetes, 2001. **50**(1): p. 18-23.

11. Trappe, S., et al., *Single muscle fiber function with concurrent exercise or nutrition countermeasures during 60 days of bed rest in women.* J Appl Physiol (1985), 2007. **103**(4): p. 1242-50.

12. Vinci, P., et al., *Early lean mass sparing effect of high-protein diet with excess leucine during long-term bed rest in women.* Front Nutr, 2022. **9**: p. 976818.

13. Brauns, K., et al., *Effects of two months of bed rest and antioxidant supplementation on attentional processing.* Cortex; a journal devoted to the study of the nervous system and behavior, 2021. **141**: p. 81‐93.

14. Brooks, N.E., et al., *Influence of exercise on the metabolic profile caused by 28 days of bed rest with energy deficit and amino acid supplementation in healthy men.* Int J Med Sci, 2014. **11**(12): p. 1248-57.

15. Buehlmeier, J., et al., *A combination of whey protein and potassium bicarbonate supplements during head-down-tilt bed rest: Presentation of a multidisciplinary randomized controlled trial (MEP study).* Acta Astronautica, 2014. **95**(1): p. 82-91.

16. D'Souza, R.F., et al., *Dairy Protein Supplementation Modulates the Human Skeletal Muscle microRNA Response to Lower Limb Immobilization.* Molecular Nutrition and Food Research, 2018. **62**(7).

17. Eijnde, B.O., et al., *AMP kinase expression and activity in human skeletal muscle: Effects of immobilization, retraining, and creatine supplementation.* Journal of Applied Physiology, 2005. **98**(4): p. 1228-1233.

18. Hendrickson, N.R., et al., *Conditionally Essential Amino Acid Supplementation Reduces Postoperative Complications and Muscle Wasting After Fracture Fixation A Randomized Controlled Trial.* Journal of Bone and Joint Surgery-American Volume, 2022. **104**(9): p. 759-766.

19. Miotto, P.M., et al., *Supplementation with dietary ω-3 mitigates immobilization-induced reductions in skeletal muscle mitochondrial respiration in young women.* FASEB journal, 2019. **33**(7): p. 8232‐8240.

20. Zeng, N., et al., *Daily protein supplementation attenuates immobilization-induced blunting of postabsorptive muscle mTORC1 activation in middle-aged men.* American Journal of Physiology - Cell Physiology, 2021. **320**(4): p. C591-C601.

21. Biolo, G., et al., *Alkalinization with potassium bicarbonate improves glutathione status and protein kinetics in young volunteers during 21-day bed rest.* Clinical Nutrition, 2019. **38**(2): p. 652-659.

22. Björkman, M., A. Sorva, and R. Tilvis, *Vitamin D supplementation has no major effect on pain or pain behavior in bedridden geriatric patients with advanced dementia.* Aging Clinical & Experimental Research, 2008. **20**(4): p. 316-321.

23. Cholewa, J.M., et al., *Dietary proteins and amino acids in the control of the muscle mass during immobilization and aging: role of the MPS response.* Amino Acids, 2017. **49**(5): p. 811-820.

24. Drummond, M.J., et al., *Bed rest impairs skeletal muscle amino acid transporter expression, mTORC1 signaling, and protein synthesis in response to essential amino acids in older adults.* Am J Physiol Endocrinol Metab, 2012. **302**(9): p. E1113-22.

25. Gao, R., et al., *The Effect of a Low Glycemic Index Pulse-Based Diet on Insulin Sensitivity, Insulin Resistance, Bone Resorption and Cardiovascular Risk Factors during Bed Rest.* Nutrients, 2019. **11**(9): p. 2012.

26. Gliemann, L., et al., *The Impact of Lower Limb Immobilization and Rehabilitation on Angiogenic Proteins and Capillarization in Skeletal Muscle.* Medicine & Science in Sports & Exercise, 2021. **53**(9): p. 1797-1806.

27. Hsieh, L.C., et al., *Effect of beta-hydroxy-beta-methylbutyrate on protein metabolism in bed-ridden elderly receiving tube feeding.* Asia Pacific Journal of Clinical Nutrition, 2010. **19**(2): p. 200-208.

28. Mose, M., et al., *Anabolic effects of oral leucine-rich protein with and without beta-hydroxybutyrate on muscle protein metabolism in a novel clinical model of systemic inflammation-a randomized crossover trial.* American Journal of Clinical Nutrition, 2021. **114**(3): p. 1159-1172.

29. Reidy, P.T., et al., *Neuromuscular electrical stimulation and protein during bed rest increases CD11b(+) skeletal muscle macrophages but does not correspond to muscle size or insulin sensitivity.* Applied Physiology Nutrition and Metabolism, 2020. **45**(11): p. 1261-1269.

30. Waha, J.E., et al., *Effects of Exercise and Nutrition on the Coagulation System During Bedrest Immobilization.* Medicine, 2015. **94**(38): p. 1-6.

31. Arentson-Lantz, E.J., et al., *Whey protein protects muscle mass loss of older adults during 7 days of inactivity and accelerates recovery of aerobic fitness during rehabilitation.* FASEB journal, 2017. **31**(1).

32. Arentson-Lantz, E.J., et al., *Preliminary findings of the effects of leucine supplementation in bedridden older adults.* FASEB journal. Conference: experimental biology 2016, EB. San diego, CA united states. Conference start: 20160402. Conference end: 20160406. Conference publication: (var.pagings), 2016. **30**(no pagination).

33. Bostock, E., et al., *Omega-3 fatty acids and vitamin D in immobilisation: Part B- Modulation of muscle functional, vascular and activation profiles.* Journal of Nutrition, Health & Aging, 2017. **21**(1): p. 59-66.

34. Johnston, A.P.W., et al., *Effect of creatine supplementation during cast-induced immobilization on the preservation of muscle mass, strength, and endurance.* Journal of Strength and Conditioning Research, 2009. **23**(1): p. 116-120.

35. Wall, B.T., et al., *The Impact of Disuse and High-Fat Overfeeding on Forearm Muscle Amino Acid Metabolism in Humans.* J Clin Endocrinol Metab, 2020. **105**(7).

36. Fransen, J.C., et al., *Impact of creatine on muscle performance and phosphagen stores after immobilization.* European journal of applied physiology, 2015. **115**(9): p. 1877‐1886.

37. Blottner, A., et al., *Whey protein plus bicarbonate supplement has little effects on structural atrophy and proteolysis marker immunopatterns in skeletal muscle disuse during 21 days of bed rest.* Journal of Musculoskeletal Neuronal Interactions, 2014. **14**(4): p. 432-444.
