# Supplementary material for: Do dietary supplements prevent loss of muscle mass and strength during muscle disuse? A systematic review and meta-analysis of randomized controlled trials
Source: Front Nutr. 2023 May 11;10:1093988. doi: 10.3389/fnut.2023.1093988 (PMC10210142; doi:10.3389/fnut.2023.1093988)
Supplement: Supplementary file 3 [file Table_3.docx]

Table S3: Methodological quality evaluated according to the PEDro scale

| **study** | **1** | **2** | **3** | **4** | **5** | **6** | **7** | **8** | **9** | **10** | **11** | **Total score** |
| --- | --- | --- | --- | --- | --- | --- | --- | --- | --- | --- | --- | --- |
| Arentson-Lantz, E. J. et al. (2020) | yes | yes | no | yes | yes | yes | no | yes | yes | yes | yes | 8/10 |
| Arentson-Lantz, E. J. et al. (2019) | yes | no | no | yes | yes | yes | yes | yes | yes | yes | yes | 8/10 |
| Backx, E. M. P. et al. (2018) | yes | yes | no | yes | yes | yes | yes | yes | yes | yes | yes | 9/10 |
| Backx, Evelien. et al. (2017) | yes | yes | no | yes | no | no | yes | yes | yes | yes | yes | 7/10 |
| Bosutti, A. et al. (2016) | yes | yes | no | yes | no | no | yes | yes | yes | yes | yes | 7/10 |
| Deutz, N. E. et al. (2013) | Yes | yes | yes | yes | yes | yes | yes | no | yes | yes | yes | 9/10 |
| Dirks, M. L. et al. (2014) | yes | yes | no | yes | no | no | no | yes | yes | yes | yes | 6/10 |
| Edwards, S. J. et al. (2020) | yes | yes | no | yes | yes | yes | yes | yes | yes | yes | yes | 9/10 |
| English, K. L. et al. (2016) | yes | yes | no | yes | yes | yes | yes | yes | yes | yes | yes | 9/10 |
| Ferrando, A. A. et al. (2010) | yes | no | no | yes | no | no | no | yes | yes | yes | yes | 5/10 |
| Fitts, R. H. et al. (2007) | yes | no | no | yes | no | no | no | yes | yes | yes | yes | 5/10 |

| Hespel, P. et al. (2001) | yes | yes | no | yes | yes | yes | yes | yes | yes | yes | yes | 9/10 |
| --- | --- | --- | --- | --- | --- | --- | --- | --- | --- | --- | --- | --- |
| Holloway, T. M. (2019) | yes | yes | no | yes | yes | yes | no | yes | yes | yes | yes | 8/10 |
| Kilroe, Sean Paul. et al. (2021) | yes | yes | yes | yes | no | no | no | yes | yes | yes | yes | 7/10 |
| McGlory, C. et al. (2019) | yes | yes | yes | yes | yes | yes | yes | yes | yes | yes | yes | 10/10 |
| Mitchell, C. J. et al. (2018) | yes | yes | yes | yes | yes | yes | yes | yes | yes | yes | yes | 10/10 |
| Paddon-Jones, D. et al. (2005) | yes | yes | no | yes | no | no | no | yes | yes | yes | yes | 6/10 |
| Paddon-Jones, D. et al. (2004) | yes | no | no | yes | no | no | no | yes | yes | yes | yes | 5/10 |
| Rudwill, F. et al. (2018) | yes | no | no | yes | no | no | no | yes | yes | yes | yes | 5/10 |
| Trappe, T. A. et al. (2007) | yes | no | no | yes | no | no | no | yes | yes | yes | yes | 5/10 |
| Frequency | 13 | 13 | 3 | 13 | 7 | 79 | 9 | 13 | 13 | 13 | 13 |  |
