# Supplementary figures and images for: Do dietary supplements prevent loss of muscle mass and strength during muscle disuse? A systematic review and meta-analysis of randomized controlled trials
Source: Front Nutr. 2023 May 11;10:1093988. doi: 10.3389/fnut.2023.1093988 (PMC10210142; doi:10.3389/fnut.2023.1093988)

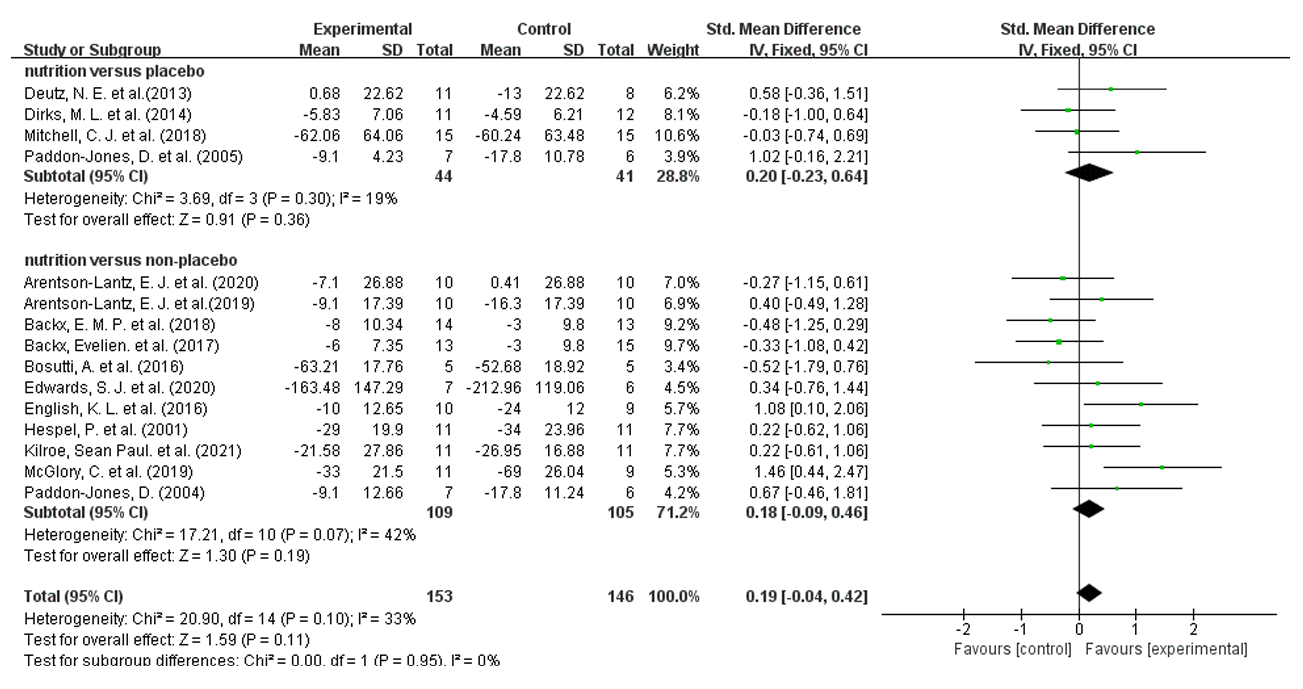

Supplement: Supplementary Figure S1 — Forest plot of subgroup analysis of SMD difference and 95% confidence intervals for the effect of dietary supplements on muscle strength according to the type of control group. [file Image_1.TIF]

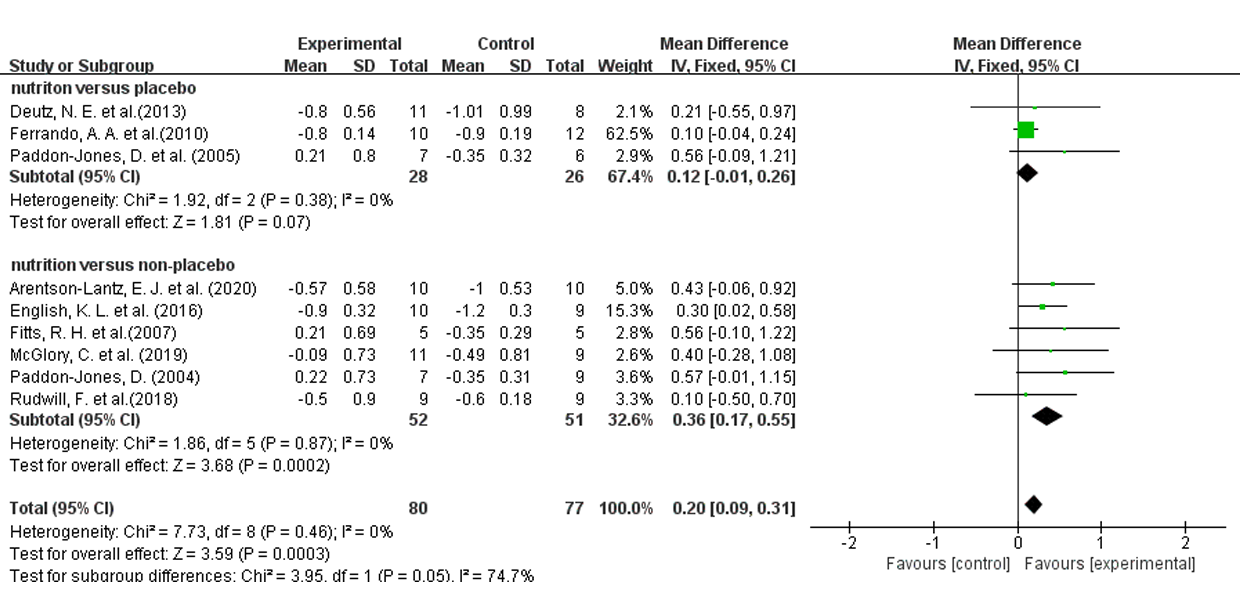

Supplement: Supplementary Figure S2 — Forest plot of subgroup analysis of SMD difference and 95% confidence intervals for the effect of dietary supplements on leg lean mass according to the type of control group. [file Image_2.TIF]

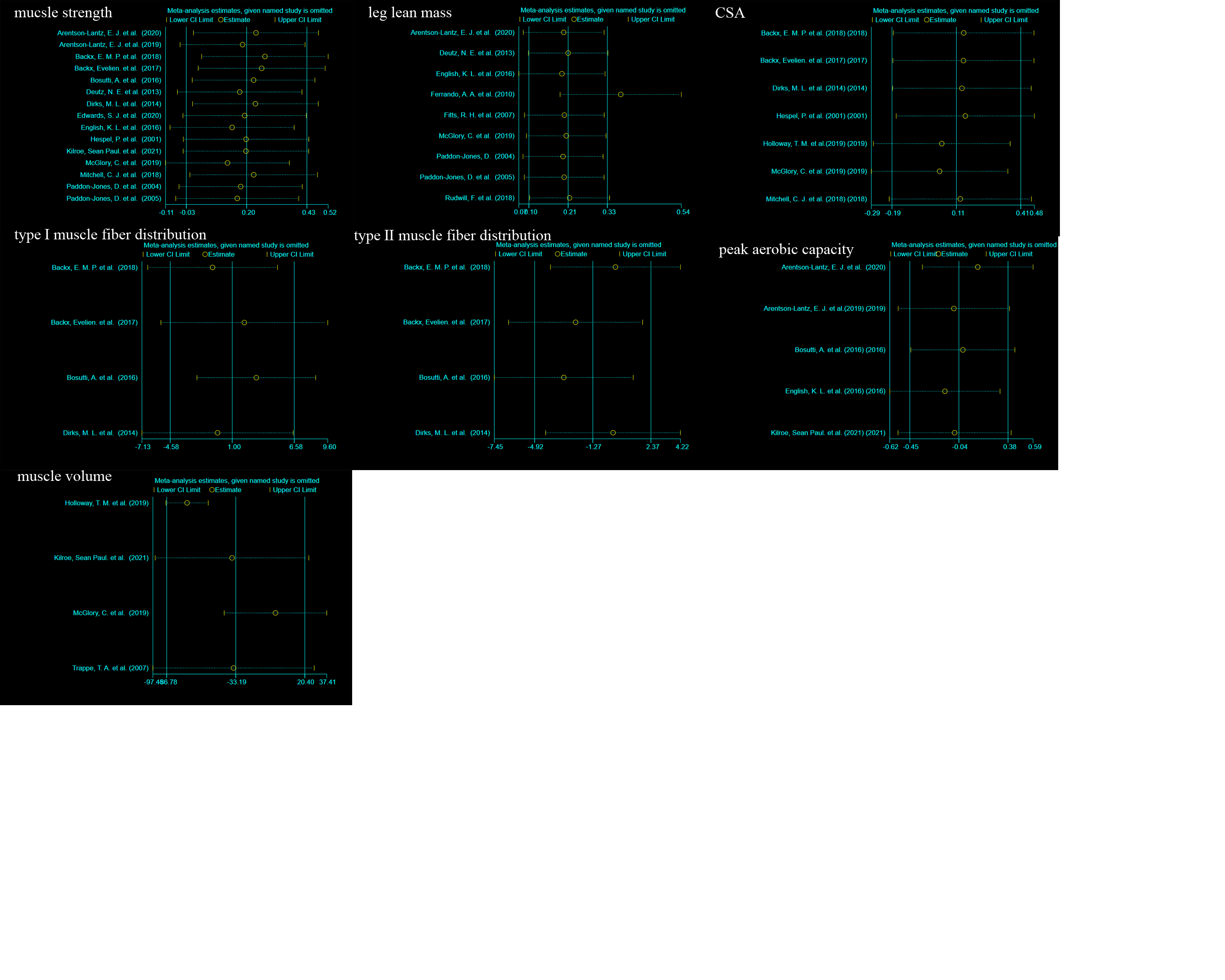

Supplement: Supplementary Figure S3 — Results of sensitivity analysis on muscle strength, leg lean mass, CSA, type I muscle fiber distribution (%), and type II muscle fiber distribution (%), peak aerobic capacity, and muscle volume. [file Image_3.TIF]
